# Transplant Trial Watch

**DOI:** 10.3389/ti.2023.12256

**Published:** 2023-11-13

**Authors:** John M. O’Callaghan, Simon R. Knight

**Affiliations:** ^1^ University Hospitals Coventry & Warwickshire, Coventry, United Kingdom; ^2^ Centre for Evidence in Transplantation, Nuffield Department of Surgical Sciences, University of Oxford, Oxford, United Kingdom; ^3^ Oxford Transplant Centre, Churchill Hospital, Oxford, United Kingdom

**Keywords:** solid organ transplant, liver transplant, deceased donor, randomised controlled trial, reperfusion

To keep the transplantation community informed about recently published level 1 evidence in organ transplantation ESOT and the Centre for Evidence in Transplantation have developed the Transplant Trial Watch. The Transplant Trial Watch is a monthly overview of 10 new randomised controlled trials (RCTs) and systematic reviews. This page of Transplant International offers commentaries on methodological issues and clinical implications on two articles of particular interest from the CET Transplant Trial Watch monthly selection. For all high quality evidence in solid organ transplantation, visit the Transplant Library: www.transplantlibrary.com.

RANDOMISED CONTROLLED TRIAL 1 Results of a Multicenter Cluster-Randomized Controlled Clinical Trial Testing the Effectiveness of a Bioinformatics-Enabled Pharmacist Intervention in Transplant Recipients.

*by Taber, D. J., et al. American Journal of Transplantation 2023 [record in progress].*

## Aims

This study aimed to report the outcomes of the cluster-randomised ISTEP trial, which aimed to examine the effectiveness of a bioinformatics-driven dashboard to guide pharmacist-led medication therapy management intervention in solid organ transplant recipients.

## Interventions

Participants were randomised to either standard care combined with the pharmacist-led, bioinformatics dashboard intervention or standard care alone.

## Participants

1982 veterans receiving 2196 transplants.

## Outcomes

The primary endpoints were the overall rate of veterans affairs (VA) emergency department (ED) visits and VA hospitalisations. Secondary endpoints included patient survival, graft survival and acute rejection episodes.

## Follow-Up

24 months.

## CET Conclusion

This interesting study from the US randomised 10 VA transplant centres, at a centre level, to use of a computerised alert dashboard designed to identify recipients at risk of non-adherence, drug interactions and abnormal/missing lab values. The authors found that use of the dashboard significantly reduced the incidence of hospital admissions (by 12.3%) and emergency department visits (by 11.3%), although the incidence of registry-reported acute rejection episodes was increased. There are potential issues with cluster randomisation in this type of study. When the number of centres is small, cluster randomisation can lead to imbalances in the groups in terms of baseline demographics and standard care levels. There is some evidence of this—ED visits and hospitalisations differed significantly in the year preceding the study between the control and intervention groups, and there are demographic and transplant mix differences as well. All of these may affect the risk of the outcomes. It is likely that the intervention was not used optimally by the participating pharmacists, with delays in responding to alerts and a lack of response to many. The key to successful implementation is therefore likely to be in optimising the workflow to ensure that alerts are acted upon in a timely fashion to achieve maximum benefit.

## Trial Registration

ClinicalTrials.gov—NCT03860818.

## Funding Source

Non-industry funded.

RANDOMISED CONTROLLED TRIAL 2 Prophylactic terlipressin infusion for severe postreperfusion syndrome in patients undergoing deceased donor liver transplantation. The TIPS-DDLT randomized controlled trial.

*by Zhang, L., et al. International Journal of Surgery 2023 [record in progress].*

## Aims

The aim of this study was to assess the effect of prophylactic terlipressin on the incidence of severe postreperfusion syndrome (PRS) in deceased donor liver transplant recipients.

## Interventions

Participants were randomised to receive either terlipressin or placebo immediately following portal vein (PV) clamping.

## Participants

64 patient scheduled for deceased donor liver transplantation.

## Outcomes

The primary endpoint was the occcurence of severe PRS after PV declamping. The secondary endpoints were hemodynamic effects following the start of the trial medication infusion, PV flow velocity after reperfusion, use of renal replacement therapy (RRT), acute kidney injury (AKI), initial poor graft function (IPGF), reoperation, and in-hospital mortality.

## Follow-Up

Not reported.

## CET Conclusion

This is an interesting randomised controlled trial in deceased donor liver transplantation. The study was small (64 patients), but adequately powered for the primary outcome of severe post-reperfusion syndrome. The study was double-blinded so that patients and clinicians were not aware of the treatment allocation. Following portal vein clamping, the study or control infusion was given at 100 mL over 10 min. The study showed a startling significant reduction in severe post-reperfusion syndrome (9% versus 53%) when using terlipressin. There was a significant difference whether using the Peking definition, van Rijn, Kork or Hilmi definition of post-reperfusion syndrome. The use of terlipressin was also associated with reduced vasopressor requirement, reduced peak ALT, and better early graft function. ICU and hospital stay were unaffected. Of concern, terlipressin was associated with increased pulmonary capillary wedge pressure and duration of mechanical ventilation. Other vasopressors were not administered prior to reperfusion so it is not clear if it is purely prophylactic action that is important, rather than terlipressin compared to other vasopressors.

## Jadad Score

5.

## Data Analysis

Strict intention-to-treat analysis.

## Allocation Concealment

Yes.

## Trial Registration

ChiCTR1800019952.

## Funding Source

Non-industry funded.

## Clinical Impact Summary

This is a well-written report of an interesting study in deceased donor liver transplantation. The trial was adequately randomised and good steps were taken to blind clinicians to the group allocation through the use of identical infusion bags. Given the trial was double-blinded in this way, one should have faith in the objective outcomes that are recorded; the primary outcome being severe reperfusion syndrome. The study was adequately powered for this outcome, defined by Peking criteria including severe/persistent hypotension during the early reperfusion period, new-onset vasoplegia during the late reperfusion period, or prolonged vasopressor treatment at the end of the surgery. Terlipressin 1 mg or placebo was administered immediately after portal vein clamping.

The trial identified a very significant reduction in the rate of severe reperfusion syndrome with the prophylactic use of terlipressin (9% versus 53%), accompanied by a significant reduction in vasopressor requirement, poor early graft function, and post-operative peak ALT. There was no difference in acute kidney injury or in-hospital mortality.

Of concern, terlipressin was associated with increased pulmonary capillary wedge pressure at 5 min after reperfusion, but this had settled by 2 h later. Mechanical ventilation was longer following terlipressin, but only by 1 h on average. These 2 issues do raise the concern for intensive monitoring for potential cardiorespiratory complications following terlipressin administration. The other fundamental concern is whether this study has identified a benefit of prophylactic pretreatment with vasopressor, or if the effect is specific to terlipressin compared to other vasopressors.

The findings of this study are in concordance with prior work done in live donor liver transplantation going back over 10 years.

